# Prevalence of domestic violence and associated factors among married women in a semi-rural area of western Turkey

**DOI:** 10.12669/pjms.305.5504

**Published:** 2014

**Authors:** Mehmet Enes Gokler, Didem Arslantas, Alaettin Unsal

**Affiliations:** 1 Mehmet Enes Gokler, Research Assistant, Deparment of Public Health, Medical Faculty, Eskisehir Osmangazi University, Eskisehir, Tukrey.; 2 Didem Arslantas, Associate Professor, Deparment of Public Health, Medical Faculty, Eskisehir Osmangazi University, Eskisehir, Tukrey.; 3 Alaettin Unsal, Professor, Deparment of Public Health, Medical Faculty, Eskisehir Osmangazi University, Eskisehir, Tukrey.

**Keywords:** Domestic violence, Prevalence, Rural areas, Turkey

## Abstract

***Objective*** ***:*** To determine the prevalence of domestic violence and associated factors among married women in a semi-rural area of western Turkey.

***Methods:*** This descriptive study was conducted between March 1 and April 29, 2011 on married women aged 15-49 years. Exposure to at least one of these types of violence at least one time within the past one year was regarded as the presence of domestic violence. Chi-square test and Logistic Regression analysis was used for statistical analysis.

**Results: **Prevalence of domestic violence against women was found to be 39.0%. About 38,4% and 26.8% of women reported verbal and psychological violence respectively. The risk factors found for the domestic violence included youngest age group, an educational level of secondary/high school for men, form of the first marriage, number of children, alcohol and gambling habits of the husband.

**Conclusion:** Our study found higher prevalence of domestic violence than expected. Verbal violence is also a significant problem particularly in terms of its consequences. It was concluded that further informative studies are needed on domestic violence to find out the causative factors to chalk out preventive strategies.

## INTRODUCTION

Violence, in fact, is a great problem that exists since the mankind exists, but since 1990, violence against women, especially domestic violence, has increased in both developed and developing countries, and has become a major public health problem.^1^ The United Nations Declaration on the Elimination of Violence against Women in 1993 defines the violence against women as “any act of gender-based violence that results in, or is likely to result in, physical, sexual or psychological harm or suffering to women, including threats of such acts, coercion or arbitrary deprivation of liberty, whether occurring in public or in private life”.^2^

The prevalence of domestic violence at any time in their life is ranging from 4% to 54% in a study conducted in 11 countries by WHO and it has been reported to range between 32.9% and 61.4% for Turkey.^3^^-^^5^

There are several factors that may be associated with domestic violence against women which can be grouped as individual factors, the factors that may be relevant to the relationship and those relevant to intimates and social norms. The factors that have been found to be associated with domestic violence against women include the education level and economic freedom of women, presence of social support, and history of domestic violence during childhood. The factors related to men include the communication level with their wives, the male-dominated society, the physically stronger nature of men, presence of alcohol or drug use, unsatisfactory income level, and witnessing domestic violence to their mothers during childhood.^6^^-^^9^

This study was designed to determine the prevalence of domestic violence against women, potentially associated factors among married women aged 15-49 years and residing in the districts of Mahmudiye and Alpu.

## METHODS

This is a descriptive study conducted between March 1 and April 29, 2011 on married women aged 15-49 years and residing in the town center of Mahmudiye and Alpu, the two districts of the province Eskisehir. According to the data of Family Health Center of each districts, total population of 15-49 age group woman was 1006 in Alpu and 1100 in Mahmudiye.

Minimum sample size for this study was 747 subjects (40.0% incidence of the event, 4% margin of error and confidence level of 95%). The study group was limited to a total of 800 women (400 women from Alpu and 400 from Mahmudiye).

The questionnaire form included questions related to sociodemographic and marital characteristics (age, education level, employment status, family income level, family type, the form of first marriage, age at first marriage, number of marriages, and number of children), addictions of their husbands (smoking status, alcohol addiction and gambling), the history of domestic violence and types of violence.^6^^,^^8^^-^^10^

Permission for the study was obtained prior to collection of data by contacting and receiving approval from the appropriate management authority, the health directorship of the city involved. Informed consent was obtained from the subjects participating in the study according to established Ethical Principles for Medical Research Involving Human Subjects in the Helsinki Declaration.

Women included in the study group were those living in the randomly selected households in the district and only one woman from each household was included to the study. After obtaining informed verbal consent, the questionnaire form was filled out by the researchers by face-to-face interview with the women found at their home during the study group and who agreed to participate in the study.

In our study, slapping, kicking, punching, pulling hair, twisting the arms, squeezing the throat and injuring with cutting or piercing instruments were defined as *physical violence*, insulting, swearing and declaiming were defined as *verbal violence*, keeping the woman without money, asking the account of expenditures and not allowing the woman to work were defined as *economic violence*, rude behaviors, being despised, humiliating and puting pressure on the woman about the persons she meets were defined as *psychological violence*, and forcing to have sexual intercourse and exposing to sexually degrading or humiliating acts were defined as *sexual violence*.^11^ Exposure to at least one of these types of violence at least one time within the past one year was regarded as the presence of domestic violence.

Individuals who smoke at least one cigarette daily were defined as smokers and those consume at least 30 grams of ethyl alcohol weekly were defined as alcohol consumers.

All data were analyzed using SPSS (version15.0) statistical package program. Chi-square test and Logistic Regression analysis was used for statistical analysis. Statistical significance was accepted at the level of p ≤ 0.05.

## RESULTS

The age of the study group ranged between 18 and 49 years and the mean age was 36.85 ± 8:44 years. In this study, the prevalence of domestic violence against women was found to be 39.0% (n = 312). The distribution of women with and without a history of domestic violence according to some socio-demographic characteristics is given in Table-I.

**Table-I:** The distribution of women with and without a history of domestic violence according to some socio-demographic characteristics

***Socio-demographic characteristics***	***History of domestic violence***			***Test value*** ***X*** ^2^ ***; p***
	*No* *n (%)* *	*Yes* *n (%)* *	*Total* *n (%)* **	
***Settlement***				
Alpu	241 (60.3)	159 (39.8)	400 (50.0)	0.189; 0.664
Mahmudiye	247 (61.8)	153 (38.3)	400 (50.0)	
***Age group***				
>25	49 (62.0)	30 (38.0)	79 (9.9)	1.103; 0.954
25-29	61 (59.8)	41 (40.2)	102 (12.8)	
30-34	80 (58.8)	56 (41.2)	136 (17.0)	
35-39	79 (61.2)	50 (38.8)	129 (16.1)	
40-44	96 (59.6)	65 (40.4)	161 (20.1)	
45-49	123 (63.6)	70 (36.4)	193 (24.1)	
***Education level of women***				
Under primary school	51 (49.0)	53 (51.0)	104 (13.0)	22.180; 0.000
Primary school	201 (58.3)	144 (41.7)	345 (43.1)	
Secondary-High school	189 (63.9)	107 (36.1)	296 (37.0)	
University	47 (85.5)	8 (14.5)	55 (6.9)	
***Educational level of husband***				
Under primary school	14 (45.2)	17 (54.8)	31 (3.9)	26.068; 0.000
Primary school	168 (59.2)	116 (40.8)	284 (35.5)	
Secondary/High school	232 (58.3)	166 (41.7)	398 (49.8)	
University	74 (85.1)	13 (14.9)	87 (10.9)	
Employment status				
Unemployed	386 (59.1)	267 (40.9)	653 (81.6)	5.326; 0.021
Employed	102 (69.4)	45 (30.6)	147 (18.4)	
***Family income level***				
Low	35 (44.9)	43 (55.1)	78 (9.8)	22.402; 0.000
Moderate	317 (58.9)	221 (41.1)	538 (67.2)	
High	136 (73.9)	48 (26.1)	184 (23.0)	
***Family type***				
Nuclear	416 (63.6)	238 (36.4)	654 (81.8)	10.250; 0.001
Patriarchal	72 (49.3)	74 (50.7)	146 (18.2)	
Total	488 (61.0)	312 (39.0)	800 (100.0)	

* Percent for the row,

** Percent for the column.

The age of the women at first marriage ranged between 13 and 35 years, with a mean age of 19.52±2.92 years. The distribution of women with and without a history of domestic violence according to some factors related to marriage and addictions of husbands is given in Table-II. 

**Table-II:** The distribution of women with and without a history of domestic violence according to some factors related to marriage and addictions of husbands

***Factors related to marrying/marriage***	***History of domestic violence***			***Test value*** ***X*** ^2^ ***; p***
	***No*** ***n (%)*** *	***Yes*** ***n (%)*** *	***Total*** ***n (%)*** **	
***First marriage***				
Arranged marriage	239 (55.1)	195 (44.9)	434 (54.3)	24.041; 0.000
Marriage by mutual agreement	231 (70.9)	95 (29.1)	326 (40.8)	
Marriage by eloping	18 (45.0)	22 (55.0)	40 (5.0)	
***The age at first marriage***				
>18	98 (51.9)	91 (48.1)	189 (23.6)	15.909; 0.003
18	84 (61.3)	53 (38.7)	137 (17.1)	
19	79 (56.4)	61 (43.6)	140 (17.5)	
20	93 (64.6)	51 (35.4)	144 (18.0)	
21	134 (70.5)	56 (29.5)	190 (23.8)	
***Number of marriages***				
The first marriage	463 (62.2)	281 (37.8)	744 (93.0)	6.053; 0.014
2	25 (44.6)	31 (55.4)	56 (7.0)	
***Number of children***				
0	28 (65.1)	15 (34.9)	43 (5.4)	12.185; 0.016
1	81 (16.6)	30 (27.0)	111 (13.9)	
2	188 (62.3)	114 (37.7)	302 (37.8)	
3	113 (57.4)	84 (42.6)	197 (24.6)	
4 or over	78 (53.1)	69 (46.9)	147 (18.4)	
***Smoking status***				
No-smoker	203 (71.5)	81 (28.5)	284 (35.5)	20.323; 0.000
Smoker	285 (55.2)	231 (44.8)	516 (64.5)	
***Alcohol consumption***				
No	432 (66.0)	223 (34.0)	655 (81.9)	37.283; 0.000
Yes	56 (38.6)	89 (61.4)	145 (18.1)	
***Habit of gambling***				
No	484 (62.1)	296 (37.9)	780 (97.5)	12.781; 0.000
Yes	4 (20.0)	16 (80.0)	20 (2.5)	
Total	488 (61.0)	312 (39.0)	800 (100.0)

* Percent for the row,

** Percent for the column

Of domestic violence against women, exposure to the verbal violence (38.4%) and sexual violence (6.9%) were reported to be the most common and the least type of violence, respectively. The distribution of women according to the type of domestic violence they were exposed is given in Table-III.

**Table-III:** The distribution of women according to the type of domestic violence

***Type of domestic violence***	***n*** *	***%***
Physical violence	66	9.8
Verbal violence	259	38.4
Economical violence	122	18.1
Psychological violence	181	26.8
Sexual violence	47	6.9
**Total**	675	100.0

* :It was assessed according to the number of domestic violence types.

The results of logistic regression analysis performed by the variables including age group, educational level, employment status, family income level, family type, the form of first marriage, age at first marriage, number of marriages, number of children, smoking-alcohol consumption of husband and gambling habit of the husband, all of which are regarded to be associated with the domestic violence against women are given in Table-IV.

**Table-IV:** Significant independent variables for domestic violence according to Logistic regression analysis

	***Model 1*** ***OR*** a *** (95% CI*** b ***)***	***Model 2*** ***OR*** a *** (95% CI*** b ***)***	***Model 3*** ***OR*** a *** (95% CI*** b ***)***
Age group	0,94 (0,85-1,03)	0,86 (0,77-0,97)	0,86 (0,77-0,97)
Educational level of woman	0,70 (0,56-0,88)	0,81 (0,64-1,03)	0,79 (0,62-1,01)
Educational level of husband	1,40 (1,12-1,75)	1,38 (1,10-1,73)	1,32 (1,05-1,67)
Employment status	0,98 (0,64-1,49)	0,93 (0,61-1,44)	1,03 (0,66-1,61)
Family income level	1,51 (1,13-2,01)	1,41 (1,05-1,90)	1,29 (0,95-1,76)
Family type	1,54 (1,06-2,24)	1,38 (0,94-2,02)	1,33 (0,89-1,98)
Form of the first marriage	-	1,65 (1,25-2,18)	1,60 (1,20-2,13)
The age at first marriage	-	1,05 (0,94-1,17)	1,03 (0,92-1,15)
Number of marriages	-	1,40 (0,78-2,51)	1,27 (0,69-2,31)
Number of children	-	1,15 (0,98-1,34)	1,19 (1,01-1,40)
Smoking status of husband	-	-	1,40 (0,99-1,99)
Alcohol consumption of husband	-	-	2,49 (1,65-3,75)
Gambling habit of husband	-	-	3,58 (1,11-11,58)

a OR : Odd’s ratio,

b CI: Confidence interval.

## DISCUSSION

Prevalence of domestic violence against women in our study was 39.0%. According to the World Health Organization (WHO) report, the prevalence ranges between 15% (Japan) and 70% (Ethiopia and Peru). These values represent the prevalence of physical and sexual violence exerted by the spouse. The prevalence of domestic violence in Turkey varies a wide range.^3^^-^^5^ Statistics on the prevalence indicate that domestic violence is a worldwide epidemic.

Increased level of education is generally associated with a high possibility of having a paying business and a better economic level. In the traditional Turkish culture, woman is the family member performing housework and man works outside and is the breadwinner person. This is still widely accepted by the inhabitants of rural areas. As long as the breadwinner is the husband, women are economically dependent on men. The possibility of having a paying business increases with the increasing level of education of women and the prevalence of domestic violence decreases among women with economic independence The low educational level among women residing in rural areas of Turkey may be considered as a factor that increases the prevalence of violence.

The prevalence of domestic violence was significantly lower among women whose first marriage was by mutual agreement (p<0.05). In the traditional Turkish culture, the spouses have no chance to know each other in arranged marriages and the woman who had never appealed her father has to continue their life without being able to appeal to her husband. Because of the fact that women with arranged marriage are mostly those without economic freedom, they may not overcome the violence.

The prevalence of domestic violence was significantly lower in women with one child compared to those without children or those with 2 or more children (p<0.05). It is an important concept in traditional Turkish society to have a child for the continuation of marriage and maintenance of the man’s descendant. In addition, increasing number of children results in economic burden especially for the families living in the rural areas, leading to increased stress at home. As a result, the prevalence of domestic violence can be expected to increase with the increasing number of children. Kocacik et al.^12^ have reported that women with no children are those exposing to least domestic violence and they have also emphasized that the prevalence of domestic violence increases 6.5 times in families with 7-8 children.

Consumption of alcohol by the husband increases the prevalence of domestic violence against the women by 2.49-fold (p<0.001). Excessive consumption of alcohol and other substances has also been considered as a factor that provokes aggressive and violent male behavior towards the wife and children. Jewkens R and colleagues^13^ have reported increased prevalence of domestic violence associated with the alcohol consumption of the spouse. On the other hand, Uskun and colleagues^14^ have found no association between alcohol consumption and prevalence of domestic violence.

The results of logistic regression model showed that the husband’s habit of gambling is an important risk factor for domestic violence (OR: 3.58; p<0.001). In a study from San Francisco, the prevalence of domestic violence against women has been found to increase 27 times if the score of the husband was 10 or above in the habit of gambling scale.^15^

The most and least common types of domestic violence against women included in the study group were verbal violence (38.4%) and sexual violence (6.9%), respectively. In Turkey, 35% of women expose to physical violence by their husband at least once throughout their life. On the other hand the rate of exposure to sexual violence has been reported to be 15%.^16^ The low prevalence of sexual violence found in this study may be related to the fact that sexual events are still a taboo that is considered as shame in the Turkish society.

## CONCLUSION

The prevalence of domestic violence against women was high (39.0%) in our study. The risk factors found in this study for the prevalence of domestic violence include youngest age group, an educational level of secondary/high school for men, form of the first marriage, number of children and alcohol and gambling habits of the husband. It was concluded that raising the awareness related to the domestic violence against women and its types and carrying out informative studies about the prevention of domestic violence are important. 
